# The Influence of Structured Nature Experiences on Youth Mental, Emotional, and Social Health Competencies in Summer Camps: A Systematic Review

**DOI:** 10.3390/bs16020246

**Published:** 2026-02-09

**Authors:** Daniela Berry, Alexandra Skrocki, Emily Howell, Daniel Pilgreen

**Affiliations:** 1 Department of Health & Wellness Design, Indiana University, Bloomington, IN 47405, USA; 2 Department of Parks, Recreation and Tourism Management, Clemson University, Clemson, SC 29634, USA; 3 Department of Recreation, Park & Tourism Sciences, Texas A&M University, College Station, TX 77843, USA; 4 Department of Rangeland, Wildlife and Fisheries Management, Texas A&M University, College Station, TX 77843, USA

**Keywords:** structured nature experiences, mental, emotional, social health competencies, summer camp

## Abstract

There is a lack of consensus on the role of structured nature experiences (SNEs) in mental, emotional, and social health (MESH) competencies across youth summer camp participants. This systematic review synthesized research on the relationship between SNEs and MESH competencies in camps since the emergence of positive youth development. Following a Google Scholar search, 21 articles were reviewed and synthesized. The findings revealed that SNEs consistently support growth in MESH competencies, particularly through experiential learning and nature immersion. The mental health domain was the most prominent MESH domain studied in the reviewed literature. Interrelated themes are discussed within each domain. While some improvements show short-term gains, lasting improvements were also observed, especially among youth with social or behavioral challenges. This review identifies SNEs as intentional and evidence-based mechanisms for advancing MESH outcomes among youth in the summer camp setting.

## 1. Introduction

Each summer, between 8 million (NASEM, 2019) and 20 million (the New York Times (Gay, 2022) citing the American Camp Association) American youth attend summer camp programs across the country. Many of these camps develop programs in line with positive youth development (PYD) characteristics and outcomes (Henderson et al., 2007; Wilson et al., 2019). As a result, common participant outcomes include increased social, physical, and cognitive skills, self-confidence, positive self-identity, independence, and leadership skills. Such outcomes are acknowledged by a broad variety of stakeholders, including campers, parents and guardians, staff members, leaders and directors, and school personnel upon returning to the classroom setting (Bialeschki et al., 2002, 2007; Henderson et al., 2007; Thurber et al., 2007).

While camper outcomes resulting from camp participation are well established in the literature, less attention is given to how specific activities or attributes may aid in growth for participants. Often, campers participate in a wide range of program offerings, such as aquatic activities, paddle sports, organized sports, climbing activities, equine sports, and large and small group games, making it difficult to discern the connection between specific activities and outcomes (Bialeschki et al., 2007). Inherently, due to the nature of these camps, many of these activities happen in an outdoor context, and camp programs rely on the natural environment to help facilitate participant growth (Dresner & Gill, 1994; Garner et al., 2015). These structured nature experiences (SNEs) during camps may provide insight into the role of nature and nature-based activities in fostering positive outcomes for campers, yet their influence remains underexplored.

We define SNEs as intentionally designed or programmed activities with learning objectives, instruction, and intended outcomes that occur outside. This definition is in line with Rossman and Schlatter’s (2015) conceptualization of structured experiences as a designed encounter wherein participants co-create the experience. SNEs can include activities such as low-ropes courses, skill-building workshops, and other guided group activities. Participation in SNEs is known to bring numerous positive participant outcomes, including improved cognitive functioning, interpersonal skills, and self-conceptualization (Bratman et al., 2015; Gill, 2014). For youth in particular, it is well established that outdoor experiences, including SNEs, improve mental health and emotional regulation (Gill, 2014; Spielvogel et al., 2024).

Many SNE outcomes are congruent with the growing body of work surrounding mental, emotional, and social health (MESH), particularly within the camp context. As youth re-entered programming following the COVID-19 pandemic, many camp professionals recognized the growing number of campers who were in need of additional MESH support (Garst et al., 2024b). In response, camps have begun to focus staff training efforts, integrate key resources, and prioritize strategies to better identify, understand, and respond to the MESH needs of both campers and staff (Garst et al., 2024a; R. Lubeznik-Warner et al., 2025; Owens et al., 2021). This shift reflects a broader trend within the youth-serving context. Recent camp literature, researchers, and practitioners are positioning camps not only as a setting for PYD, but also as critical environments for promoting MESH growth and well-being (R. P. Lubeznik-Warner & Rosen, 2023; Tan et al., 2024).

MESH outcomes in the camp setting are multifaceted and commonly explored as a collective that encompasses a range of competencies and indicators. These include self-esteem, sense of purpose, resilience, and social acceptance (Orth et al., 2022). Within SNEs, these competencies are often viewed as foundational for fostering safe environments where youth feel supported, understood, and empowered to participate (Vasilaki et al., 2025).

This systematic review sought to synthesize research on the relationship between SNEs and MESH competencies in camps since the emergence of PYD in the academic literature, in turn, serving to inform camp practice and drive a holistic approach to future MESH/SNE camp research.

## 2. Methods

Our systematic review was informed by Xiao and Watson’s (2019) guidelines for systematic literature reviews. In these guidelines, they divide literature reviews based on their purpose. Given our goals of synthesizing research on the connection between SNEs and MESH outcomes in camps, the purpose of the present review is to describe the literature, and most closely aligns with a textual narrative synthesis (Xiao & Watson, 2019). Textual narrative synthesis is an approach to systematic literature reviews that employs a standardized data extraction format that allows for the examination of relationships within and between studies (Popay et al., 2006). The primary data extracted were camp characteristics, presence of SNEs, and MESH outcomes. Additionally, textual narrative syntheses often categorize studies to allow description of the literature within and across relevant categorical boundaries (Xiao & Watson, 2019). During data synthesis, we categorized studies based on the examined MESH domain(s) and present the findings through these domains. The authors’ positionality and process of data extraction, coding, and synthesis are explained in greater detail below.

### 2.1. Positionality

Because textual narrative reviews involve interpretive judgement, we offer the following positionality statement to contextualize the perspectives of the research team that inform this review. All team members are White, were raised in the United States, and have participated in or worked at youth camps in the past. Our team is guided by pragmatic and post-positivist orientations, drawing primarily on quantitative methods supplemented with qualitative methods. Our individual research experience with camp contexts and MESH outcomes varies but is collectively robust. These methodological and experiential perspectives informed our synthesis of the literature.

### 2.2. Search Strategy

The literature search was conducted using the Google Scholar database, as this is the main free resource providing access to scholarly articles for practitioners. For instance, a podcast for camp professionals, The Pudding, encourages its listeners to use Google Scholar to improve camp programming because they can “search the way your brain is used to” (Baker & Allison, 2024). While the decision to only use Google Scholar does have its limitations, it is supported by research on database coverage within the systematic review methodology (Gehanno et al., 2013).

It is not currently possible to export search results on Google Scholar, and manually extracting pertinent information through scraping methods is difficult because of Google Scholar’s tendency to block IP addresses after as few as ten searches. However, the software program Publish or Perish (Harzing, 2007) attempts to remove this barrier by providing a point-and-click application, which can return up to 1000 results per search from Google Scholar in a format that can be exported for further analysis. These results mimic those a practitioner would see when using Google Scholar themselves. Therefore, the Publish or Perish v.8 software was used to search the literature.

Search terms were developed using the Population, Concept, Context (PCC) framework (Pollock et al., 2023). Following this framework, multiple search strings were developed involving youth as the population, MESH outcomes and SNE as the concepts, and camp as the context. In the search software, a keyword search is equivalent to a standard Google Scholar search. The software reads “|” as “OR” and spaces as “AND”. The final keyword search, informed by these PCC terms and the character limit, was:

*Youth|child|camper “summer camp”|“resident camp”|“day camp”|“overnight camp” nature|outdoor|“outdoor experience”|“experiential learning” “social competence”|“social intelligence”|“emotional intelligence”|“emotional competence”|“interpersonal skills”*

Since PYD emerged in the literature in 1999 (Hamilton, 1999), it has been overwhelmingly adopted as the dominant youth development framework informing youth programming in the United States (Arnold & Silliman, 2017; Catalano et al., 2004). As such, the minimum year field was set to 1999, following the emergence of PYD in the youth development literature, and all other search fields in the search software were left blank. To ensure that all relevant research was captured, included studies were not limited to a particular methodological approach.

### 2.3. Eligibility Criteria

Eligibility criteria were scaffolded from title review to full-text review, increasing in stringency to satisfy the aims of the review. To pass the initial title screenings, article titles must have included a reference to any type of camp (e.g., camp, day camp, overnight camp, summer camp) *or* nature element (e.g., outdoors, nature, wildland) *and* a reference to the youth life stage (e.g., youth, child, girls/boys) *or* mention a MESH outcome or MESH outcomes broadly (e.g., resilience, self-esteem, social health). Thus, a title mentioning youth in the outdoors would be included for the abstract review; however, a title mentioning only a nature-based camp would not be included. Articles were excluded at this stage if their titles mentioned countries outside of the US and Canada or used the word “student” to refer to their population. These exclusions were an effort to ensure the review focused on out-of-school time programs, rather than outdoor-based academic or wilderness therapy programs. This same process was utilized for abstract screening (i.e., must include camp or nature element plus youth or MESH outcome).

During the full-text review, articles were included in the final review if they met all of the following eligibility criteria:

- Study was conducted at camp,
- Study was conducted with campers about their self-reported experiences,
- The article named at least one SNE that occurred at camp (e.g., ropes course, kayaking, hiking, etc.),
- The study included at least one MESH outcome, and
- The article was broadly informed by theory.

Articles were excluded from final review if they met any of the following exclusion criteria:

- Full text not available in English,
- Study was conducted outside of the United States or Canada,
- Study did not take place in the outdoors,
- The only SNE mentioned was arts and crafts, organized sport, or physical activity,
- Data were collected from students within a school setting,
- Data were retrospective (i.e., more than 18 months removed from their camp experience), or
- Article was a systematic review of the literature.

### 2.4. Literature Selection and Extraction

Literature was extracted from Google Scholar in May 2024 using Publish or Perish (Harzing, 2007) with maximum results set to 1000. The initial search in Google Scholar yielded 988 articles. All results were exported from Publish or Perish and uploaded into GoogleSheets where the researchers conducted each stage of the screening. After articles not written in English (*n* = 4) were removed, the remaining 984 articles were advanced for title screening. To establish inter-rater reliability (IRR; McHugh, 2012) across the four-person research team, a subset of 199 (~20%) articles was independently reviewed by all four team members, using the established criteria. Each team member indicated whether or not the article should be included. The research team then discussed any articles with inclusion misalignment and re-coded as necessary. The IRR for this subset was 93% and supported dividing the remaining articles (*n* = 785) equally across the team members for title screening. Article titles were each reviewed by two reviewers (IRR = 87%). In instances where two reviewers did not agree, a third reviewer served as the tiebreaker. Reasons for exclusion at the title review stage included the absence of camp and/or MESH outcomes, inclusion of students, or articles not based in the US or Canada. Following the title review, the remaining 112 articles underwent abstract review. IRR was re-established for the abstract review with a subset of 20 articles and resulted in an IRR of 82%. Following the same procedures as utilized within the title review, the remaining articles (*n* = 92) were then divided across the research team, and each abstract was reviewed by two reviewers. Reasons for exclusion at the abstract review stage mirrored the title phase (e.g., absence of camp and/or MESH outcomes, inclusion of students, or articles not based in the US or Canada).

**Figure 1:**
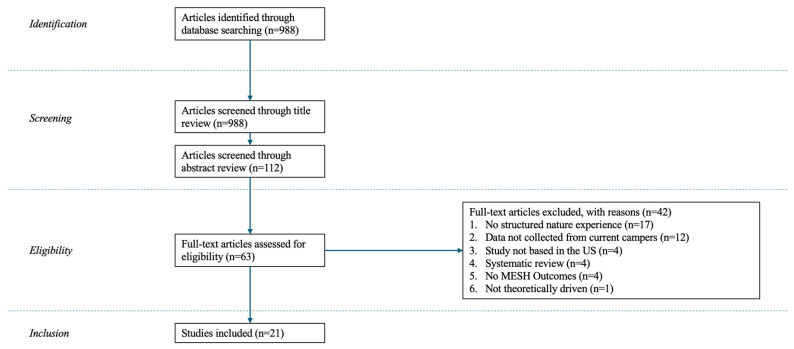
PRISMA diagram depicting the process from search to inclusion.

Following the abstract review, 63 articles were advanced for full-text review and data extraction. Given the depth and breadth of the included articles, data extraction concentrated on study purpose, incorporated theories or frameworks, selected methods, sample size and camp characteristics, findings, and suggestions for future research and/or practice. To establish extraction expectations, a subset of 12 articles was reviewed together as a research team. Once consensus was reached, the remaining articles (*n* = 51) were then divided for full-text review and data extraction by one member of the research team. Each extraction record was then reviewed by two reviewers. Following full-text review and extraction, 21 articles were selected for review inclusion and synthesis. The two most salient reasons for exclusion at the full text and data extraction stage included (1) no mention of SNEs (*n* = 17) and (2) data were not collected directly from campers (*n* = 12). See Figure 1 for a visualization of our data reduction process.

### 2.5. Data Synthesis

Following data extraction, reviewed manuscripts were grouped within MESH domains based on the presence of domain indicators in the manuscripts’ dependent variables identified during data extraction. Manuscript grouping was not mutually exclusive, and a manuscript could be synthesized in multiple domains. For example, Faith et al. (2019) reported dependent variables in both the mental and emotional health domains; therefore, their findings were included for synthesis in both groups.

Within each domain, the authors examined the primary findings identified during data extraction and inductively identified commonalities across studies to develop emergent themes. This process was guided by thematic synthesis (Thomas & Harden, 2008) in that it involved moving from study-level findings to higher-order themes that captured patterns of evidence. Themes were then iteratively compared against the detailed findings, extracted as verbatim paragraphs from the manuscripts’ results sections, and the full manuscripts to ensure accuracy and grounding in the reported evidence. This constant comparison process also provided specific supporting evidence for each theme, ensuring that themes were both representative of the overall domain and demonstrably supported by individual studies. Conclusions were drawn from the literature based on the number of studies supporting the conclusion. Refinements to themes and conclusions were made through reviewer consensus.

## 3. Results

A comprehensive description of included articles is presented in Appendix A Table A1. The 21 articles in our sample were distributed across 19 years of publication. Although the publication date in the search was constrained to 1999 in keeping with the establishment of the tripart ideation of PYD (Hamilton, 1999), the first paper included in our sample was not published until 2005. The publishing trend by year is stable across three decades, with slightly more publications per year in the 2010s. Articles were published by a variety of disciplinary journals, including the *Journal of Park and Recreation Administration*, *Journal of Pediatric Nursing*, and *Child and Adolescent Social Work Journal*.

The articles in our sample have a substantial overlap in their stated aim or purpose. Of the 21 articles, 18 (85.7%) were focused on how program participation is related to one or more MESH outcomes. Five papers (23.8%) investigated whether a specific program characteristic was related to MESH outcomes. These specific characteristics ranged from a phone-free environment (Uhls et al., 2014) to free choice in daily activities (Schmalz et al., 2011). Two papers (9.5%; Martin, 2018; Uhls et al., 2014) structured their analysis from a program evaluation lens wherein improved MESH outcomes for campers translated to a programmatic success. Only one paper’s aim was to expand upon an existing theory by testing it within a camp setting (Mishna, 2005). Half of the included articles employed PYD as their theoretical framework, citing foundational works including Eccles and Gootman (2002) and Lerner (2002). Within the remaining articles, there was no one dominant theory, rather a collection of psychological theories (e.g., hope theory (Snyder, 1995); self-determination theory (Ryan & Deci, 2000)) and developmental theories (e.g., developmental systems theory (Lerner & Castellino, 2002); social cognitive learning theory (Bandura, 1997)) were adopted. However, across all articles, there were inconsistencies in the depth of theory application. For example, a large portion of papers merely referenced the theory in their introduction, whereas others provided clear connections to the cited theory throughout the entirety of their article. While acknowledgement of theory was required for final inclusion, these studies present a wide range of theory integration.

### 3.1. Synthesized Themes

Findings from the included studies were thematically synthesized by MESH domain. Specific but interrelated themes were created for each domain, with the mental health domain as the most prominent MESH domain studied (*n* = 18; 85.7%). The social health domain was the second most represented domain (*n* = 16; 76.2%) in our collection of studies, followed by emotional health (*n* = 8; 38.1%). While fewer studies explored specific social and emotional health outcomes, findings from the studies largely overlap with these domains. The synthesized findings for each MESH domain and their respective themes are presented below.

### 3.2. Mental Health

Three subthemes were identified when synthesizing findings from the 18 (85.7%) papers that reported mental health outcomes of SNEs in youth camp: self-esteem and self-perception, sense of purpose, and engagement. While these themes were distinct within the literature, they are also interrelated and often provide evidence for multiple themes within one finding.

#### 3.2.1. Self-Esteem and Self-Perception

A preponderance of the evidence within this domain supports a connection between youth camps that leverage SNEs and self-esteem, self-perception, and perceptions of competence. Patterns across studies suggest that campers often report increased self-esteem, personal confidence, and positive self-perception during and following their participation in camps. Rimmer et al. (2007) found that campers with burns and those without burns reported no significant difference in self-esteem post-camp despite those with burns having significantly lower self-esteem prior to their second year at a medical specialty camp focusing on youth with severe burns. Similarly, results reported by Devine and Dawson (2010) indicate that campers’ self-esteem significantly increased from before to after camp through a pre- and post-test at an overnight camp for those with craniofacial differences. Their findings showed no significant decline in self-esteem at a six-month post-camp follow-up assessment.

Conversely, Arnoldo et al. (2006) found that the majority of campers at a specialty burn camp reported no significant change in self-esteem. However, taken together, the majority of the quantitative evidence from the domain supports a positive relationship between camp experience and self-esteem. Qualitative evidence further supports this conclusion and provides additional insight. Evidence presented by Schmalz et al. (2011) further suggests that the process of activity selection during SNEs at camp is an important mechanism for connecting experiences to a sense of self. For instance, a participant “expressed that finding her own activity based on interests and competence supports her understanding of herself, and her self-worth” (Schmalz et al., 2011, p. 61). Beyond autonomy in the decision process, evidence also points to overcoming challenges associated with nature experiences as a driver for developing a sense of self (Schmalz et al., 2011). For example, within the camp industry, “challenge by choice” is a widely adopted best practice that emphasizes the importance of challenge while allowing participants autonomy to determine their level of engagement. Within this framework, evidence suggests increased participation and positive outcomes for attendees (Chase, 2014/2015). These findings, in turn, indicate sustained improvements in self-esteem among individuals who attend camps with SNEs.

Beyond self-esteem, multiple studies (*n* = 7) reported that camp was associated with an increased sense of self, identity development, and self-worth. Chawla and MacDermid Wadsworth (2012) found that a one-week overnight camp for children and youth with parents in the military significantly increased children’s self-worth. Qualitative evidence from an environmental education camp for urban youth indicates that “the program was a catalyst for increased self-esteem” (p. 73) and gave campers “a heightened sense of independence and confidence” (Roberts & Suren, 2010, p. 72). Aligning with these findings, Campbell et al. (2022) found that camp-based interventions, including SNEs specifically, significantly increased self-perceptions in youth who had been victimized, and Browne et al. (2011) found that nature-based camp experiences significantly improved campers’ self-reported independence and empowerment.

#### 3.2.2. Sense of Purpose

The reviewed studies provided consistent empirical evidence of the positive relationship connections between SNEs at camp and campers’ sense of purpose. A thematic analysis of camper experiences by Roberts and Suren (2010) found that campers were motivated by self-directed tasks and challenging outdoor activities. Further, campers suggested that they were “making discoveries about themselves and their identities” (Roberts & Suren, 2010, p. 73). Supporting the role of SNEs in developing a sense of purpose, Linver et al. (2023) found that campers who attended high-adventure camps reported significantly higher levels of sense of purpose than those who participated in a summer camp that was not high-adventure and those who did not participate in summer camp at all. These findings provide qualitative and quantitative evidence of the influence of SNEs on campers’ sense of purpose.

Adjacent concepts such as hope, conceptualized by Snyder (1995) as a psychological strength that insulates youth from adversity consequences, can be an essential component of emerging purpose and goal-orientation. Findings from Hellman and Gwinn (2017) found that participation in an overnight camp, including SNEs, significantly improved hope scores in pre- and post-tests of adolescents exposed to domestic violence. Further, their results suggest a positive association between hope and increased perseverance, optimism, and curiosity. Similarly, Whittington et al. (2016) found that girls participating in an outdoor adventure camp reported significant increases in optimism and sense of mastery from pre- to post-camp, and that increases persisted one month after participation. Qualitative results from Griswold et al. (2014) indicate that campers often used the words “hope” and “optimistic” to describe the camper–-counselor relationship at a summer camp for youth with Tourette’s syndrome. Conversely, findings from Faith et al. (2019) found no statistically significant difference in self-reported hope between pre- and post-camp participation in an overnight medical specialty camp. However, they did find a significant and positive relationship between the number of activities participated in and hope scores post-camp, linking nature experiences at camp with these concepts. Although adjacent, hope, optimism, and mastery can support the development of a greater sense of purpose and life satisfaction (Cotton Bronk et al., 2009). Together, these findings provide evidence that supports the notion that camps with SNEs nurture the development of a sense of purpose and goal directedness in campers.

#### 3.2.3. Engagement

A number of qualitative and mixed-method studies (*n* = 6) identified a connection between camps that employed SNEs and camper engagement, often through experiential and hands-on learning. For instance, the camp programming offered in Griswold et al. (2014) was considered to be unique and memorable by campers and led to opportunities for activity participation and mental challenge that facilitated camper relationships and personal growth. Aligning with these findings, Burns and colleagues (Burns et al., 2023) found that an adventure STEM camp was effective in fostering task-specific confidence related to outdoor recreation. Their results reflected sustained mental focus and engagement in STEM-related outdoor tasks, which built camper confidence. Similarly, Son et al. (2017) presented evidence that participation in an outdoor adventure education STEM program provided a sense of competence through “opportunities to learn novel, relevant information; the unique outdoor learning environment; a highly engaged instructional approach; and opportunities to feel successful” (p. 38). Beyond this, program participants noted that the natural environment and being outside positively impacted engagement and learning (Son et al., 2017). Schmalz et al. (2011) found similar results in that challenges associated with nature experiences led to novel problem solving and a sense of accomplishment. Browne et al. (2011) also revealed a significant increase in campers’ self-reported problem-solving confidence before and after a nature-based camp. Finally, qualitative and quantitative evidence from Linver et al. (2023) triangulate to suggest day and overnight camp experiences at Boy Scouts of America camps provide “a novel physical and social environment where youths have the opportunity to grow” (p. 1294) and that high-adventure programs specifically “facilitate the cultivation of goal-oriented skills by providing opportunities to reach new challenging goals in novel environments” (p. 1294). Together, these findings generally support that camps with SNEs stimulate mental involvement by creating experiential learning and encouraging active participation and curiosity and consistently describe engagement as an outcome of camp experiences.

### 3.3. Emotional Health

Across the 21 papers selected for inclusion, 8 (38.1%) reported emotional health-related outcomes. Campers’ emotional health was divided into two themes: emotional regulation and resilience. Similar to mental health themes, emotional regulation is related to resilience. The literature reviewed sometimes uses emotional regulation and resilience interchangeably; however, we synthesized the data based on operationalization and descriptions of the concepts, not necessarily the nomenclature used within the research.

#### 3.3.1. Emotional Regulation

The body of literature reviewed provided evidence that camps with SNEs support youth in regulating their emotions and improving reactivity in stressful situations. Allen et al. (2011) found that campers at a two-week day camp for youth at risk for behavioral and academic problems developed stronger emotional awareness and self-control. Campers reported statistically significant increases in their reported ability to deal with their own anger in response to a disparate situation from after camp relative to before. Campers also reported increases in their frequency of thinking before acting and controlling their own temper in response, but differences did not reach statistical significance. In findings reported by Whittington et al. (2016), participants at an all-girls adventure camp felt “better able to effectively cope with novelty and challenge” (p. 9) and less emotionally reactive after participating in camp. Specifically, the authors note that campers reported being less sensitive to stressors and less likely to be overwhelmed by their emotions (Whittington et al., 2016). Importantly, differences were reported in nearly all campers who participated in the adventure camp, suggesting that the adventure camp experience was a facilitating factor in these changes. Whittington and colleagues (Whittington et al., 2016) propose that the opportunities to step outside of comfort zones and challenges afforded by adventure-based and outdoor camps are important components of camp for emotional health. Findings from Gillard and Allsop (2016) corroborate this notion in relation to the camp experience at an overnight medical specialty camp. One camper reported that, without camp, they think they “would be a very angry person” (Gillard & Allsop, 2016, p. 117) and that camp improved their overall positive affect.

Other evidence from this literature ties camp participation to emotional regulation, but is less supportive of the connection between nature experience and emotional regulation. Further, self-perceptions of behavioral control increased from pre-camp to 1-month post camp across all participants, and continued to increase for four months post camp for one of the three case studies (Campbell et al., 2022). Authors attribute these emotional and behavioral improvements to campers’ connection to counselors and counselors supporting appropriate responses to stressful situations. For instance, one camper experienced teasing while at camp and counselors “encouraged her to create positive statements about herself and distance herself from challenging peers” (Campbell et al., 2022, p. 578), highlighting the role of camp counselors in supporting emotional reactivity. Alternatively, findings from Hellman and Gwinn (2017) present evidence from an overnight camp that connects hope and self-control, including ability to regulate thoughts, feelings, and behaviors. These findings, when coupled with the results of Faith et al. (2019), link participation in activities at camp with hope and the ability to regulate thoughts and emotions. Together, these findings support emotional regulation as an outcome of structured nature experiences during youth camp participation; however, the mechanism by which participation duration influences regulation is still unclear and warrants additional investigation.

#### 3.3.2. Resilience

Multiple studies (*n* = 7) examined the connection between the camp experience and campers’ perceptions of hope and resilience. Many of the studies support a positive relationship between camp and resilience. For instance, Allen et al. (2011) found that campers showed increases in their perceived ability to handle peer pressure, avoid at-risk behaviors, and stay out of fights, although these increases did not meet the threshold for statistical significance. For participants in an adventure-based camp for girls, self-reported resilience was higher post-camp relative to pre-camp and persisted in a one-month-post-camp follow-up assessment (Whittington et al., 2016). The persistence of the effect of adventure camp on resilience beyond the camp experience is especially notable given that the experience consists of only five days, highlighting the potential for nature-based experiences at camp to improve emotional outcomes in youth.

### 3.4. Social Health

Social health outcomes were the second most widely noted outcomes of camps with SNEs, with 16 of the 21 papers (76.2%) selected for full-text review reporting social-health-related outcomes. Three social health themes were identified from the results of these 16 studies: social belonging, communication and relationship skills, and social development. These themes highlight the ways in which camps can foster meaningful peer interactions and support overall social growth within SNE’s.

#### 3.4.1. Social Belonging

Results from the reviewed literature offer both quantitative and qualitative evidence that supports a positive relationship between camp experiences and youths’ sense of social belonging and acceptance. Chawla and MacDermid Wadsworth (2012) found that campers’ perception of peer acceptance, popularity, and likability significantly increased following a camp-based intervention relative to pre-camp in youth who have parents in the military. Devine et al. (2015) also found significant differences in perceived social acceptance pre- and post-camp for youth with hearing difficulties. Aligning with these findings, Whittington et al. (2016) found that girls who participated in an outdoor adventure camp reported that they were more comfortable interacting with others after they participated in the camp, and Gillard and Allsop (2016) found that sense of belonging was the most prominent outcome for campers at a medical specialty camp. Similarly, Devine and Dawson (2010) provide evidence from a camp for youth with craniofacial differences, that campers experienced significant improvements in their perceptions of social acceptance. However, these increases were considered to be temporary and were not observed in post-camp follow-up assessments six to eight weeks after camp. Evidence from a focus group of campers with Tourette’s syndrome suggests that camp facilitated engagement that reduced feelings of isolation associated with their disability and increased feelings of social inclusiveness (Griswold et al., 2014). These improvements in social belonging were often tied to the recreational nature of the camp environment; Griswold et al. (2014) state “it was seen that the opportunity to share mutually exclusive space and take part in fun recreational activities together contributed to the campers’ level of social connectivity and feelings of belonging” (p. 23). Together, these findings indicate that SNEs may build interpersonal confidence and overall sense of acceptedness in youth, although the persistence of these outcomes is uncertain.

Contrary to the findings of increased belonging and acceptance through camp experiences, Linver et al. (2023) found no statistically significant differences in cultural humility, defined as perceptions of peer acceptance of cultural differences, across youth who participated in a high-adventure camp, a non-adventure summer camp, and those who did not participate in summer camp. While these findings collectively present a mix of outcomes regarding participation in nature-based camp experiences and social belonging, the overall body of evidence points to the potential of camps to support peer connection and foster acceptance among youth.

#### 3.4.2. Communication and Relationship Skills

Multiple papers (*n* = 11) provide evidence of the effectiveness of camps at improving relationship and communication skills. In a case study of three campers that had experienced childhood victimization, quantitative self-assessments of social competence found that, for the two female campers, social competence improved significantly from pre- to one-month-post-camp and continued to increase for four months after camp (Campbell et al., 2022). Allen et al. (2011) found similar increases related to social skills, including being a good listener and using civil language, in campers’ pre- and post-camp self-perception scores at a day camp focusing on character development; however, these differences did not reach statistical significance.

The role of camp staff and the camp setting was also revealed as an important catalyst for the improvement of social skills. In a case study of a camper with learning disabilities, camp counselors worked with the camper to support her understanding of social cues and to develop social skills with her peers while attending an adventure-based camp (Mishna, 2005). The researcher credits the camp staff’s facilitation during adventure activities, noting they provide “idealizing experiences that allow the children to feel safe and able to take risks” (Mishna, 2005, p. 63), including pushing the boundaries of their social spheres. Alternatively, the cabin setting provided by many camps offers opportunities to develop and hone social skills. For instance, qualitative data from a free-choice outdoor adventure camp indicated that navigating interpersonal relationships contributed to the camp experience and that these skills “might translate to working with others in activities” (Schmalz et al., 2011, p. 62). Beyond interpersonal skills, there was one study which provided experimental evidence associated with cognitive social abilities. In an experimental study of campers who were allowed screen time during camp versus those who were not, campers who were away from their screen for the camp session were significantly better at recognizing facial emotions relative to those who had screens (Uhls et al., 2014). However, the findings were largely associated with the lack of screen time, not necessarily nature or camp experiences. While this evidence does not directly link participation in SNEs to these social experiences, they do tie camp participation to growth in social health.

Results from Linver et al. (2023), however, do tie SNEs to social outcomes through an experimental study of adventure campers, non-adventure campers, and youth who did not attend summer camp. Linver and colleagues (Linver et al., 2023) found that campers who participated in a high-adventure Boy Scouts camp reported significantly higher communication skills compared to those who attended a non-adventure camp and youth who did not attend a camp. These results clearly support the positive role of SNEs during camp in improving communication and social skills. The authors suggest this increase stems from the “comprehensive challenges that unfold in novel and outdoor settings” (Linver et al., 2023, p. 1294). Consistent with this idea, Griswold et al. (2014) reported that SNEs, such as a ropes course, were a modality for campers to engage and socialize with each other, providing a challenge through which campers overcome fears with the help of their peers. Additionally, Hellman and Gwinn (2017) found a correlation between increases in self-reported hope in a pre- and post-camp assessments and counselor-observed social intelligence. Evaluating the body of evidence collectively, there is support for a connection between the camp experience and social skills. Further, quantitative and qualitative evidence is consistent in suggesting SNEs support interpersonal skills.

#### 3.4.3. Social Development

A number of papers (*n* = 8), especially those that employed a qualitative design, illustrate that campers develop communities and friendships through their time at camp. Rimmer et al. (2007) suggest that campers with burns reported high levels of integration within the camper community of campers both with and without burns. This feeling of community within the camp setting was present across multiple years of the study, and it was true regardless of the visibility of burns, years since burn, or how long they had been at camp (Rimmer et al., 2007). Similarly, qualitative evidence presented by Martin (2018) indicates that the camp structure “nurtured a social sphere in which interpersonal connections fostered community” (p. 169). Griswold et al. (2014) suggest that the camp experience provided campers with Tourette’s syndrome an opportunity to meet others with similar disability experiences, facilitating strong relationships between campers and peer-support networks. Further, findings from Griswold and colleagues (Griswold et al., 2014) point to specific outdoor experiences, namely, the ropes course and zip line, as particularly beneficial for interpersonal bonding, and that cabin-based, camp-wide experiences, such as outdoor games and competitions, supported relationship development between campers.

## 4. Discussion

### 4.1. Key Findings

This review demonstrates strong and consistent evidence that SNEs in youth camp settings foster positive MESH outcomes. Across the 21 studies included in this review, both qualitative and quantitative findings show that SNEs can be a key mechanism for supporting camper well-being (e.g., Devine et al., 2015). Findings from the most prominent outcome, mental health, also indicate that SNEs play an important role in fostering competencies such as self-esteem, self-perception, sense of purpose, and cognitive engagement. Campers frequently reported increased confidence, improved self-worth, and a stronger understanding of personal strengths following participation in SNEs (e.g., Browne et al., 2011; Roberts & Suren, 2010). Quantitative studies documented significant gains in self-esteem across diverse populations, including youth with burns, craniofacial differences, and those who had experienced trauma (e.g., Campbell et al., 2022; Faith et al., 2019). Notably, several studies found that these improvements persisted beyond the camp session, with some reporting sustained effects at one-month follow-ups (Whittington et al., 2016). Structured nature activities that involved overcoming physical or social challenges, such as high-adventure courses or outdoor skill-building tasks, were consistently linked to these mental health outcomes (Linver et al., 2023; Schmalz et al., 2011). These findings align with experiential learning theory, which emphasizes the development of competence and self-efficacy through active participation in challenging yet supportive environments (Rossman & Schlatter, 2015).

Social health outcomes were the second most frequently reported outcomes, with campers consistently describing gains in belonging, peer connection, communication skills, and relationship development (e.g., Griswold et al., 2014; Linver et al., 2023). These outcomes were observed in both traditional and specialized (i.e., population or activity specific) camp programming, suggesting that the social benefits of SNEs are not limited to a particular type of program or population. Programs with activities such as ropes courses, outdoor adventure challenges, and group-based environmental education have noted these activities as being conducive to social growth, providing opportunities for cooperative problem-solving and shared accomplishment that align with theories of PYD and experiential learning (Schmalz et al., 2011).

Emotional health outcomes were also evident across the literature, particularly in the domains of emotional regulation and resilience. Campers described feeling better able to manage stress, cope with change, and regulate their emotions during and after camp (Gillard & Allsop, 2016). Quantitative results indicated increases in campers’ reported ability to think before acting, manage anger, and remain calm in stressful situations (Allen et al., 2011). Adventure-based activities and other SNEs that intentionally placed youth outside their comfort zones were frequently identified as key contexts for these changes (Hellman & Gwinn, 2017; Whittington et al., 2016). Several studies noted that emotional gains were often facilitated by supportive counselor relationships, suggesting that the social environment of camp interacts with nature-based experiences to produce positive emotional outcomes (Campbell et al., 2022; Gillard & Allsop, 2016).

A notable strength of this review is the diversity of camps represented in the included studies. The sample encompassed a wide range of camp types, including day and overnight programs, co-ed and single-gender camps, and medical specialty camps serving youth with conditions such as diabetes, burns, and craniofacial differences. Such diversity is uncommon in camp research, which often relies on homogenous or convenience samples (Spielvogel et al., 2024). The consistent positive outcomes across these varied contexts strengthen the generalizability of the findings and suggest that SNEs may be a universally beneficial component of the camp experience for youth. Moreover, the variety of structured nature activities identified (e.g., ropes courses, hiking, kayaking) illustrates the breadth of opportunities within camps to intentionally leverage the natural environment for youth development (Sibthorp et al., 2020).

Collectively, these findings highlight the unique role of SNEs in supporting MESH outcomes. While improvements in mental and emotional health may manifest differently across individuals and camp types, the consistent association between SNEs and enhanced social belonging underscores the social foundation of these experiences (Dopko et al., 2019; Spielvogel et al., 2024). Whether through shared challenge, collaborative learning, or immersion in nature, SNEs provide a structured yet flexible setting in which campers can develop critical competencies that support their overall well-being.

### 4.2. Limitations

Several limitations should be considered when interpreting these findings. First, the search was limited to Google Scholar, which, while practitioner-friendly and broadly inclusive, may have excluded relevant literature indexed only in other databases. Second, the use of Publish or Perish software introduced constraints related to search replication and export capabilities, which could limit the reproducibility of the process. As an interface to Google Scholar, Publish or Perish does not have privileged access to Google Scholar search results. Therefore, searches are subject to Google Scholar’s rate limiting and anti-automation mechanisms, including restrictions on repeated queries from a single IP address. Searches of this magnitude trigger protections which constrains retrieval. Additionally, Google Scholar relies on dynamic indexing and rank-based results ordering, wherein search results may vary across users and over time. Third, the lack of a clear and consistent definition of MESH across the included studies complicated synthesis and comparison. Although defining MESH was outside the scope of this review, this gap reflects a broader challenge in the literature and underscores the need for conceptual clarity in future research. Finally, only two studies employed an experimental design, which limits the ability to attribute MESH outcomes directly to structured nature experiences rather than to the overall camp environment. This limitation reflects the current state of the literature rather than the methodology of this review; however, it remains important when considering the strength of causal claims.

### 4.3. Future Directions

While this review provides strong evidence that SNEs may be correlated to campers’ MESH outcomes, future research should more precisely identify the mechanisms through which these benefits occur. A key next step is to determine whether improvements in social health stem directly from the nature-based elements of camp programming or from the broader camp experience itself. Understanding the extent to which SNEs uniquely support social belonging, communication, and relationship skills would help practitioners design programs that maximize developmental outcomes. Such research may be well suited to mixed-methods or longitudinal designs that compare varying levels of SNE exposure, incorporate multiple stakeholder perspectives, and assess changes in social belonging, communication, and relationship skills over time.

Many of the articles in this review cited in their own future directions the need for more experimental research within the camp space. Much of the camp literature relies on cross-sectional data or short-term pre–post designs, lacking both longitudinal significance and a true experimental methodology, limiting reportable causal relationships. For camps with broad program offerings, it may be beneficial to compare participants’ frequency and duration of SNEs and reported MESH outcomes. Additionally, for camps serving specific populations, it may be beneficial for future, more pointed research to explore how unique populations (e.g., diabetics, burn victims, etc.) experience SNEs in the camp environment differently. Further, differing understandings of camp SNEs across populations may inform different MESH outcomes.

As noted in the design of this review, the included studies reported only on camper data. In the future, triangulation between stakeholders could help produce a more robust understanding of the relationship between SNEs and MESH outcomes. For example, including camp staff perspectives, parent/guardian observations when campers return home, or even healthcare providers and educators, may offer a more robust understanding of campers’ self-perceptions or behavioral outcomes.

## 5. Conclusions

This systematic review provides compelling evidence that participation in camp programs with SNEs was a powerful catalyst for fostering MESH outcomes. Across 21 peer-reviewed studies spanning nearly twenty years of research, findings consistently demonstrate that SNEs, defined as intentionally designed, instructional outdoor activities, contribute meaningfully to camper growth and well-being. By integrating elements of experiential learning, cooperative challenge, and intentional reflection, SNEs serve as a bridge between nature exposure and measurable psychosocial development.

Mental health outcomes were the most prevalent across the literature, with participation in SNEs being correlated with positive mental health competencies such as self-esteem, self-efficacy, sense of purpose, and cognitive engagement. Equally important, social health outcomes highlight camps as unique environments where belonging, interpersonal connection, and communication skills are cultivated through shared outdoor experiences. Emotional health outcomes, including resilience and emotional regulation, were also strengthened when campers engaged in outdoor challenges that encouraged self-awareness, perseverance, and adaptability. Collectively, this review identifies SNEs not only as recreational or environmental components of camp, but as intentional and evidence-based mechanisms for advancing MESH outcomes among youth.

## Data Availability

No new data were created or analyzed in this study.

## Appendix A

**Table A1:** Description of Included Articles.

Study	Study Design	Study Sample	Population Served	Geographic Location	Camp Characteristics	Structured Nature Experiences	MESH Domains and Measures ^ab^	Major Findings
(Allen et al., 2011)	Quantitative Pre-/Post-test	50	Coed, At risk youth with behavioral and academic problems	Virginia, US	Day Camp 1 Site 2 Weeks	Adventure climbing challenge	M, E, S Character development (DeRoche, 2004)	Growth in camper reported outcomes of caring, helpfulness, and anger management
(Arnoldo et al., 2006)	Quantitative Pre-/Post-test	45	Coed, Youth burn survivors	Texas, US	Overnight 1 Site 1 Week	Nature hikes, fishing, canoeing	M Rosenberg Self-Esteem Scale (1989)	Reported scores on self-esteem scale varied for burn camp participants with 29% increasing. 13% decreasing, and 58% reporting no change Not enough statistical support that this particular program enhanced self-esteem on chosen scale
(Browne et al., 2011)	Quantitative Quasi-experimental; Pre-/Post-test	177	Coed	US	Day and Overnight 13 Sites M = 23.8 days	Nature based problem solving, interactive nature experiences	M Leadership Skills—(ACA Youth Outcomes Battery, YOB; Sibthorp et al., 2010), Civic Engagement for Youth Scale (Flanagan et al., 2007)	Growth in camper reported independence, problem-solving, affinity for nature, and empowerment
(Burns et al., 2023)	Qualitative Semi-structured interviews	15	Coed	West Virginia, US	Overnight 1 Site 8 Days	Geology	M self-efficacy	Adventure stem program was effective in establishing and improving perceptions about outdoor task-specific confidence
(Campbell et al., 2022)	Mixed Methods Pre-/Post-test and follow-up	3	Coed, youth who have experienced childhood victimization	Not reported	Overnight 1 Site 1 Week	Campfire, field games	M, E, S Strengths and Difficulties Questionnaire (Goodman, 1997); Victimization (JVQR2-RIV; Hamby et al., 2011)	Programming was effective in increasing self-perception for youth who had been previously victimized
(Chawla & MacDermid Wadsworth, 2012)	Quantitative Pre-/Post-test	44	Coed, Military dependents	Indiana, US	Overnight 1 Site 1 Week	Outdoor activities and games that develop an understanding of the local fauna and wildlife	M, E, S Self Perception Profile for Children (SPPC; Harter, 1985); Self-Perception Profile for Adolescents (SPPA; Harter, 1988)	One-week programs can positively impact camper’s social acceptance, athletic competence, and global self-worth
(Devine & Dawson, 2010)	Quantitative Longitudinal, 3 time points	31	Coed, Youth with craniofacial differences	Midwest, US	Overnight 1 Site 1 Week	Fishing, swimming, climbing, teambuilding, cabin campouts, recreational games, archery, arts, music, and nature activities	M, S Rosenberg Self-Esteem Scale (Rosenberg, 1965, 1988); Social Acceptance/Perception (source not listed)	Reported self-esteem was higher at the post-test than the pre-test, 6-week follow up was not statistically different that post-test Reported higher social acceptance at post-test than pre-test, 6-week follow up was not statistically different than pre- or post-test
(Devine et al., 2015)	Quantitative Longitudinal, 3 time points	46	Coed, Youth with cochlear implants or hearing aids	Midwest, US	Overnight 1 Site 1 Week	Hiking, swimming, horseback riding, sports, nature, climbing, canoeing, adventure therapy	S Social Acceptance Scale (SAS; Devine, 1997); Peds QL General Well-Being Scale (Hallstrand et al., 2003; Varni et al., 1999)	Social Acceptance and Related Quality of Life increased between pre- and post-test and was maintained 10 weeks post camp
(Faith et al., 2019)	Quantitative Pre-/Post-test	62	Coed, Youth with cancer, sickle cell, renal disease, heart disease, other chronic conditions	Midwest, US	Overnight 1 Site 1 Week	Fishing trips; optional activities include swimming; high ropes courses	M, E Children’s Hope Scale (CHS; Snyder et al., 1997); Child Attitude Toward Illness Scale (CATIS; Austin & Huberty, 1993); Benefit and Burden Scale for Children (BBSC; Currier et al., 2009)	Positive correlation between number of activities and reported hope post-camp
(Gillard & Allsop, 2016)	Qualitative Videoed interviews	24	Coed, Children with serious illnesses	Northeast, US	Overnight 1 Site 7 Days	Outdoor activities, fishing	M, E, S belonging, enjoyment, be myself, positive affect, growth	Campers shared that camp was characterized by, sense of belonging, enjoyment, positive affect, camp programming, adult staff, personal growth, and escape
(Griswold et al., 2014)	Qualitative Focus groups and observations	18	Coed, Youth with Tourette’s	Midwest, US	Overnight 1 Site 1 Week	Swimming, boating, horseback riding, and a ropes course with a zip line	M, S relatedness, social development, programmatic outcomes	For campers with Tourette Syndrome, self-reported social outcomes included, relatedness, social assurance, social development Specific programs fostered camper bonding such as the ropes course and camp-wide games
(Hellman & Gwinn, 2017)	Quantitative Pre-/Post-test	229	Coed, Children exposed to domestic violence	US	Overnight 9 Sites 6 Days	Rafting, tubing, high and low ropes challenge courses, horseback riding, kayaking and canoeing, recreational hiking and field games, nightly campfires	M, E, S Children’s Hope Scale (CHS; Snyder et al., 1997)	Program participants reported increased hope and resilience
(Linver et al., 2023)	Mixed Methods Longitudinal surveys, 3 time points; 1 time interviews	4121 (online survey), 106 (interviews)	Coed, Youth involved with Boy Scouts	New Mexico, Florida Keys, Minnesota/Canada boundary waters, West Virginia, US	Day and Overnight 4 Sites 6-10 Days	Backpacking and hiking expeditions, snorkeling, scuba diving, sailing, and fishing, conservation efforts and marine biology studies, canoeing and winter camping expeditions, ziplining, rock climbing, mountain biking, and white water rafting.	M, E, S communication, ethical decision making, connection to others, citizenship, purpose, leadership, joy/fun, perceived cultural humility	Attendees of a high-adventure camp scored higher on communication, citizenship, and sense of purpose than those at other camps or with no camp experience High-adventure activities helped youth develop leadership skills
(Schmalz et al., 2011)	Qualitative Focus groups, one-on-one interviews	26	Girls	Vermont, US	Overnight 1 Site 4 or 8 Weeks	Can choose between 16 activity departments, at any time, for any duration.	M, S sense of self	Free choice activity opportunities gave campers a better sense of self Interpersonal skills and problem solving skills also improved for some participants.
(Son et al., 2017)	Qualitative Focus groups	22	Coed, Suburban youth	Pacific Northwest, US	Overnight 1 Site 5 Days	Outdoor adventure education, snow science course	M, S optimal engagement, self-determination, values	Participants reported noticeable differences in OAE and traditional learning environments. Course supported self-determination and ‘fun while learning’
(Uhls et al., 2014)	Quantitative Pre-/Post-test	51 (experimental), 54 (control)	Coed	California, US	Overnight	forest ecology, outdoor skills, animal survival, day hike, archery, orienteering	S The Child and Adolescent Social Perception Measure (CASP); Media use (Pea et al., 2012; Uhls, 2013)	Social interaction in nature can help participants better understand the emotions of other individuals as opposed to digital social interactions
(Whittington et al., 2016)	Quantitative Pre-/Post-test w/longitudinal subset	26 (reported on the RSCA), 87 (participated in the program)	Girls, Youth who are socially and economically disadvantaged	Vermont, US	Day Camp More than 1 Site 5 Days	Mountain biking, daily trail rides, a two-part self-defense workshop	M, E, S Resiliency Scale for Children and Adolescents (RSCA^®^); (Prince-Embury, 2007)	Program participants reported higher levels of resilience, increased skill master and relatedness, and decreased emotional reactivity

^a^ Full references of the instrument development papers can be found in the Appendix B. ^b^ M = mental health; E = emotional health; S = social health.

## Appendix B. References for Measures in Table A1

Austin, J. K., & Huberty, T. J. (1993). Development of the child attitude toward illness scale. *Journal of Pediatric Psychology*, *18*(4), 467–480. https://doi.org/10.1093/jpepsy/18.4.467.
Currier, J. M., Hermes, S., & Phipps, S. (2009). Children’s response to serious illness: Perceptions of benefit and burden in a pediatric cancer population. *Journal of Pediatric Psychology*, *34*(10), 1129–1134. https://doi.org/10.1093/jpepsy/jsp021.
DeRoche, E. (2004). *Evaluating character development: 51 tools for measuring success*. Character Development Group.
Devine, M. A. (1997). *The relationship between social acceptance and leisure participation for people with disabilities* [Unpublished dissertation]. University of Georgia.
Flanagan, C. A., Syvertsen, A. K., & Stout, M. D. (2007). *Civic engagement models: Tapping adolescents’ civic engagement*. CIRCLE Working Paper, 55. Available online: https://www.researchgate.net/publication/234700265_Civic_Measurement_Models_Tapping_Adolescents%27_Civic_Engagement_CIRCLE_Working_Paper_55 (accessed on 14 July 2025).
Goodman, R. (1997). The strengths and difficulties questionnaire: A research note. *Journal of Child Psychology and Psychiatry*, *38*(5), 581–586. https://doi.org/10.1111/j.1469-7610.1997.tb01545.x.
Hallstrand, T. S., Curtis, J. R., Aitken, M. L., & Sullivan, S. D. (2003). Quality of life in adolescents with mild asthma. *Pediatric Pulmonology*, *36*, 536–543. https://doi.org/10.1002/ppul.10395.
Hamby, S., Finkelhor, D., Turner, H., & Kracke, K. (2011). *The juvenile victimization questionnaire toolkit*. University of New Hampshire. Available online: https://www.unh.edu/ccrc/juvenile-victimization-questionnaire (accessed on 14 July, 2025).
Harter, S. (1985). *The self-perception profile for children: Revision of the Perceived competence scale for children* [Manual]. University of Denver.
Devine, S. (1988). *Manual for the self-perception profile for adolescents* [Manual]. University of Denver.
Napier, T. L., & Wright, C. J. (1974). *An evaluation of forced relocation of population due to rural community development* (Research Bulletin 1073). Ohio Agricultural Research and Development Center. Available online: https://core.ac.uk/download/pdf/159572899.pdf (accessed on 14 July 205).
Pea, R., Nass, C., Meheula, L., Rance, M., Kumar, A., Bamford, H., Nass, M., Simha, A., Stillerman, B., Yang, S., & Zhou, M. (2012). Media use, face-to-face communication, media multitasking, and social well-being among 8–12 year old girls. *Developmental Psychology*, *48*, 327–336. http://dx.doi.org/10.1037/a0027030.
Prince-Embury, S. (2007). *Resiliency scales for children and adolescents: Profiles of personal strengths manual*. Harcourt Assessments.
Rosenberg, M. (1965). *The Rosenberg self-esteem scale*. Princeton University Press. https://doi.org/10.1037/t01038-000.
Rosenberg, M. (1988). Self-objectification: Relevance for the species and society. *Sociological Forum*, *3*(4), 548–565. https://doi.org/10.1007/bf01115414.
Rosenberg, M. (1989). *Society and the adolescent self-image*. Wesleyan University Press.
Sibthorp, J., Browne, L. P., & Bialeschki, M. D. (2010). Problem-solving and camp connectedness: Two new measures for the ACA youth outcomes battery. *Research on Education in the Outdoors*, *10*, 1–12. https://doi.org/10.1353/roe.2010.0002.
Snyder, C. R., Hoza, B., Pelham, W. E., Rapoff, M., Ware, L., Danovsky, M., Highberger, H. R., & Stahl, K. J. (1997). The development and validation of the Children’s Hope scale. *Journal of Pediatric Psychology*, *22*(3), 399–421. https://doi.org/10.1093/jpepsy/22.3.399.
Uhls, Y. (2013, April 18–20). *Values and new media: How social media relates to preteen values*. The Society for Research on Child Development, Seattle, WA, USA.
Varni, J. W., Seid, M., & Kurtin, P. S. (1999). Pediatric health-related quality of life measurement technology: A guide for health care decision makes. *Journal of Clinical Outcomes Management*, *6*, 33–40.
